# Fungal disease incidence along tree diversity gradients depends on latitude in European forests

**DOI:** 10.1002/ece3.2056

**Published:** 2016-03-11

**Authors:** Diem Nguyen, Bastien Castagneyrol, Helge Bruelheide, Filippo Bussotti, Virginie Guyot, Hervé Jactel, Bogdan Jaroszewicz, Fernando Valladares, Jan Stenlid, Johanna Boberg

**Affiliations:** ^1^ Department of Forest Mycology and Plant PathologySwedish University of Agricultural SciencesBox 702675007UppsalaSweden; ^2^ BIOGECOUniversity of BordeauxUMR120233615PessacFrance; ^3^ INRABIOGECOUMR120233612CestasFrance; ^4^ Institute of Biology/Geobotany and Botanical GardenMartin Luther University Halle‐Wittenberg06108HalleGermany; ^5^ German Centre for Integrative Biodiversity Research (iDiv) Halle‐Jena‐Leipzig04103LeipzigGermany; ^6^ Department of Agricultural Food and Environmental SciencesUniversity of Firenze50144FirenzeItaly; ^7^ INRADYNAFORUMR120131326Castanet‐TolosanFrance; ^8^ Białowieża Geobotanical StationFaculty of BiologyUniversity of Warsaw17230WarsawPoland; ^9^ Museo Nacional de Ciencias NaturalesConsejo Superior de Investigaciones Cientificas28006MadridSpain

**Keywords:** Diversity gradient, Europe, foliar fungal pathogen, forests, FunDivEUROPE, latitudinal gradient, tree species richness

## Abstract

European forests host a diversity of tree species that are increasingly threatened by fungal pathogens, which may have cascading consequences for forest ecosystems and their functioning. Previous experimental studies suggest that foliar and root pathogen abundance and disease severity decrease with increasing tree species diversity, but evidences from natural forests are rare. Here, we tested whether foliar fungal disease incidence was negatively affected by tree species diversity in different forest types across Europe. We measured the foliar fungal disease incidence on 16 different tree species in 209 plots in six European countries, representing a forest‐type gradient from the Mediterranean to boreal forests. Forest plots of single species (monoculture plots) and those with different combinations of two to five tree species (mixed species plots) were compared. Specifically, we analyzed the influence of tree species richness, functional type (conifer vs. broadleaved) and phylogenetic diversity on overall fungal disease incidence. The effect of tree species richness on disease incidence varied with latitude and functional type. Disease incidence tended to increase with tree diversity, in particular in northern latitudes. Disease incidence decreased with tree species richness in conifers, but not in broadleaved trees. However, for specific damage symptoms, no tree species richness effects were observed. Although the patterns were weak, susceptibility of forests to disease appears to depend on the forest site and tree type.

## Introduction

In the context of climate change, European forests are increasingly threatened by fungal pathogens and the damage and disease they cause (Stenlid et al. 2011; Jactel et al. 2012). Over the last few decades, the number of new pathogens introduced into European forests has grown exponentially as a result of increased global trade and movement of plant material (Santini et al. 2013). Thus, the consequences of increased pathogen activity in forest ecosystems that affect the functions, services, and products have been the subject of growing concern (Crooks 2002; Fisher et al. 2012; Boyd et al. 2013). Subsequently, to better mitigate fungal pathogen impact in the future, it is important to reduce forest susceptibility to disease. Several consequences of forest management have been shown to influence stand vulnerability to pathogens, among which one of the most important is tree species diversity and composition (Jactel et al. 2009).

Diversity of species is considered beneficial for most ecosystems (Cardinale et al. 2011, 2012; Gamfeldt et al. 2015). Higher levels of ecosystem services, such as biomass production, soil carbon storage, and berry production, have been found in mixed forests with increasing number of tree species (Gamfeldt et al. 2013; Carnol et al. 2014). Biodiversity is also considered important to ecosystem stability (Tilman 1999; Jucker et al. 2014; Morin et al. 2014), and mixed forests are thought to reduce the risk of fungal pathogen disease susceptibility as compared to monospecific species stands (Pautasso et al. 2005). High tree species diversity may maintain the overall integrity of a forest ecosystem, as proposed by the insurance hypothesis (Yachi and Loreau 1999). However, the extent to which the increase in tree species richness *per se* mitigates the impact of fungal pathogens remains controversial (Koricheva et al. 2006).

Three main categories of ecological processes may explain why the diversity of neighboring tree species can affect the likelihood of contamination by, or the vulnerability to diseases of a focal tree (Barbosa et al. 2009). The first category is “numerical” and related to the relative proportion of conspecific and heterospecific neighbors (Kim and Underwood 2015). Reduction in disease risk with increasing density of heterospecific neighbors is called the dilution effect (sensu Keesing et al. 2006; Civitello et al. 2015). For instance, for fungal root pathogens, such as *Heterobasidion annosum*, disease transmission via root contacts was reduced with a decreasing concentration of susceptible hosts (Piri et al. 1990; Gerlach et al. 1997; Lindén and Vollbrecht 2002). With regard to foliar fungal pathogens, Hantsch et al. (2013) found a negative correlation between tree species richness in the local neighborhood of the target tree and the infection level of several oak powdery mildew species that was mainly brought about by higher host dilution. However, the opposite effect might be true, with a higher risk of pathogen damage with increasing density of heterospecific neighbors, as pathogens may concentrate on fewer focal host trees (i.e., host concentration effect (Root 1973)). An increase in the proportion of susceptible *Quercus* species hosts was observed to increase tree mortality due to the oak wilt pathogen *Ceratocystis fagacearum* (Menges and Loucks 1984). The other two categories of processes correspond to emerging properties of mixing different tree species, namely those related to the composition of forest mixtures. These “true” associational resistance effects can be either bottom‐up, only due to plant interactions, or top‐down, that is, involving other trophic interactions. The main “bottom‐up” processes are related to host tree apparency (Castagneyrol et al. 2014), with the presence of heterospecific neighbors reducing the probability of colonization of the focal tree. Non‐host neighboring trees may provide a physical barrier against wind‐dispersed spores, thereby reducing the probability of spores landing on the focal host tree in mixed stands (Heybroek 1982).

Hantsch et al. (2014a), while describing reduced pathogen infestation levels by local tree diversity, which were independent of the host species density, also suggested that the presence of heterospecific neighbors had modified local microclimate with adverse consequences on spore dispersal or germination. The “top‐down” associational effects result from mixed forests providing natural enemies with more habitats or feeding resources. For example, mixtures of tree species might better accommodate antagonistic fungi that slow the spread of fungal pathogens such as *H. annosum* (Johansson and Marklund 1980; Fedorov and Poleschuk 1981) and *Phytophthora cinnamomi* (Murray 1987). However, mixing tree species may also lead to higher pathogen damage, in other words, associational susceptibility. This is particularly true for heteroecious fungal pathogens that require two unrelated hosts to complete their life cycle. If both of these host tree species are present in the same mixed forest, the latter is more likely to experience damage. One example is the pine twisting rust caused by *Melampsora pinitorqua* that requires *Pinus sylvestris* and *Populus tremula* as alternate hosts (Mattila 2005).

Stand composition, the particular tree species assemblages, has been therefore suggested to be more important for reducing fungal disease than strictly the number of tree species (Setiawan et al. 2014) as already demonstrated with pest damage (e.g., Jactel and Brockerhoff 2007; Sobek et al. 2009). The functional composition is particularly important; for example, whether the associated tree species belong to the same class (i.e., broadleaved trees or conifers (Jactel and Brockerhoff 2007)). Decreased mortality of susceptible conifers by *Armillaria* root rot was observed in mixed conifer and broadleaved stand, rather than mixed conifer stands (Morrison et al. 2014). The evolutionary relatedness among plant host species, also known as the phylogenetic diversity, may also influence the total amount of disease that may result from pathogen spillover of closely related host species (Branco et al. 2015; Parker et al. 2015). Experimental inoculations of tropical trees with foliar fungal pathogens showed that the proportion of tree species that developed disease decreased with phylogenetic distance between plants (Gilbert and Webb 2007). Based on the previous studies, tree diversity thus appears to reduce fungal pathogen incidence and severity, especially if unrelated tree species are mixed. However, disease incidence will also depend on whether the pathogen is a generalist or specialist, thereby dictating their host range.

Many tree diversity studies have been conducted on local and regional scales, focusing on specific tree species or specific fungal pathogens. Yet, it is not clear whether the effect of tree species diversity on overall fungal pathogen incidence is generalizable across such a wide range of mature forest ecosystems at the continental scale, regardless of tree species, pathogen, or forest ecosystem. Furthermore, at such a large spatial scale, latitudinal patterns, which may be linked with changes in temperature and precipitation patterns, can have an effect on biotic interactions (Qian and Ricklefs 2007; Vacher et al. 2008; Kozlov et al. 2015).

In 2011, 209 long‐term monitoring plots were established in mature forests to study the functional significance of tree species diversity across a wide latitudinal gradient, constituting the FunDivEUROPE Exploratory Platform (Baeten et al. 2013). In this study, we utilized these plots to conduct our study in six major forest types across Europe, from the southern Mediterranean to the northern boreal forest biomes. We determined the incidence of foliar fungal disease on 16 focal tree species. The objective was to determine the relationship between tree species diversity and foliar fungal disease incidence across varying European forest types. Specifically, we analyzed the influence of tree species richness, tree functional type, and phylogenetic diversity on overall fungal pathogen incidence. We hypothesized that fungal incidence decreases with tree diversity across all forest types, irrespective of latitude.

## Material and Methods

### Study sites

**Figure 1:**
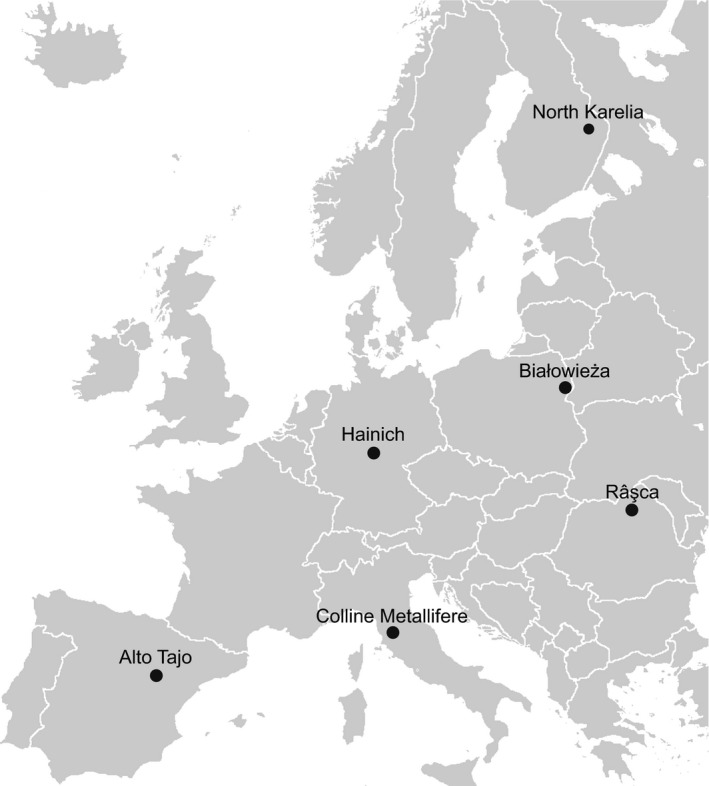
Map showing the location of the six sampling sites in the respective European country. See Table 1 for site and sampling details.

The study was conducted in six mature forest sites (at least in the late to mid‐stem exclusion stage) in six countries, spanning six major European forest types (Fig. 1, Baeten et al. 2013). Sampling was conducted over approximately 2 weeks in each of the six countries during the vegetation period (June to August), in either 2012 or 2013. The number of plots sampled varied in each site and, likewise, the time of sampling (Table 1). In total, 209 plots were sampled.

**Table 1:** Summary of sampling sites and focal tree species assessed for foliar fungal disease incidence along the latitudinal gradient in Europe

Forest Site	Alto Tajo	Colline Metallifere	Râsca	Hainich	Bialowieza	North Karelia
	Spain	Italy	Romania	Germany	Poland	Finland
Forest Type	Mediterranean mixed	Thermophilous deciduous	Mountainous beech	Beech	Hemiboreal, nemoral, coniferous, mixed broadleaved–coniferous	Boreal
Mean Forest Age (Years)	90	62	85	111	92	42
Location	40.7° N, 1.9° W	43.2° N, 11.2° E	47.3° N, 26.0° E	51.1° N, 10.5° E	52.7° N, 23.9° E	62.6° N, 29.9° E
Sampling Period	June 2013	June–July 2012	July 2013	July 2012	July–August 2013	August 2012
Plots Sampled	36	36	28	38	43	28
Max Richness Level	4	4	4	4	5	3
Focal Tree Species (Number of sampled trees per species per region)						
Broadleaved						
*Acer pseudoplatanus*			42	53		
*Betula pendula*					72	60
*Carpinus betulus*					81	
*Castanea sativa*		57				
*Fagus sylvatica*			63	92		
*Fraxinus excelsior*				72		
*Ostrya carpinifolia*		50				
*Quercus cerris*		63				
*Quercus faginea*	75					
*Quercus ilex*	51	66				
*Quercus petraea*		56		43		
*Quercus robur*				2	75	
Conifer						
*Abies alba*			51			
*Picea abies*			51	34	75	60
*Pinus sylvestris*	54				75	60
*Pinus nigra*	72					

Standardized plots of 30 × 30 m were previously established in all countries. Different compositions of tree species were targeted to create a tree species richness gradient ranging from one to three‐ (Finland), four‐ (Romania, Germany, Spain), or five‐species (Italy, Poland) mixtures and different tree species compositions at each level of species richness. Target tree species had similar relative abundances (i.e., high evenness), with a lower limit of 60% of maximum evenness based on basal area. At least two trees per species were present in the mixtures. There were four exceptions. In one plot in Germany, there was one tree each of *Acer pseudoplatanus* and of *Quercus robur*. In another plot, there was one tree of *Q. robur* and in a different plot, one tree of *Q. petraea*. The presence of non‐target tree species was kept at a minimal, which was less than 5% of the total basal area. In total, we sampled 16 focal tree species that were regionally common and/or economically important (Table 1).

### Data collection: leaf sampling and fungal disease incidence assessment

Focal trees were randomly selected from a pool of those trees with the largest diameter at breast height within plots: six trees in monoculture plots and three trees per species in mixtures. In total, 1605 trees were sampled using extension pruners, by shooting down the leaves, or with the help of tree climbers. Two branches were sampled with southern exposure: one from the sun‐exposed upper part of the canopy and one in the lower third of the canopy. Foliar samples (leaves for broadleaved species and shoots for conifer species) were collected from each sampled tree. Per branch, 25–30 (the exception being that up to 60 leaves per branch were collected in Italy) for broadleaves, or 10 current‐year shoots for conifers were sampled, resulting in a total of 50–60 leaves or 20 shoots per sampled tree.

In each plot, we recorded the occurrence of five a priori determined types of fungal pathogen damage (signs or symptoms): oak powdery mildew, leaf spots, and unknown fungal pathogen damage type for the broadleaved tree species, and rust and needle cast for the conifer species. Visual inspection for fungal pathogen damages (or suspected damages caused by fungi) was conducted on fresh leaves and shoots within 1 day of sampling. To avoid observer bias, one person (DN) conducted all assessments. The number of leaves or shoots with the respective damage types, out of the total leaves or shoots sampled, was recorded for each tree. There were instances where leaves were recorded with two types of damages, for example both leaf spots and powdery mildew, either on the same leaf or on two different leaves. Therefore, to avoid overcounting the number of damaged leaves, the total number of leaves with at least one type of damages was noted to have a tree‐level value for disease incidence.

Disease incidence per tree was defined as the number of leaves or shoots with any type of damage, regardless of the damage type relative to the number of leaves or shoots without damages. The final data set included observations from 1605 trees. In a given country and tree species richness level, the percentage of diseased leaves and shoots, regardless of tree species identity, species composition, or damage type, was determined by first calculating the mean disease incidence per tree from the number of leaves or shoots with damage and then by calculating the mean across all trees for a specific tree species richness level (see Table S1 in Supporting Information).

Additionally, the incidence of tree species‐specific diseases caused by identifiable specialist fungal pathogens was recorded for a selected subset of tree species. These pathogens occurred in specific countries. The pathogens examined here included (i) spruce needle rust caused by *Chrysomyxa ledi* on *P. abies* in Finland (*n* = 60 trees)*,* (ii) tar spot caused by *Rhytisma acerinum* on *A. pseudoplatanus* in Romania (*n* = 42), and (iii) oak powdery mildew caused by *Erysiphe* sp. in Poland (*n* = 75 *Q. robur*) and Germany (*n* = 43 *Q. petraea* and 2 *Q. robur*).

### Statistical analysis

We tested the relationship between tree species diversity and fungal disease incidence using generalized linear mixed models (GLMMs). These types of models accounted for the hierarchical structure of the data and allowed for nested and crossed random‐effect terms (Zuur et al. 2009; Schielzeth and Nakagawa 2013). We used the logarithm of the ratio of number of damaged to undamaged leaves per tree as the response variable, with a binomial error distribution. There was non‐independence among observations within each plot (several trees from the same species sampled in the same plot) and among plots within countries (several plots of the same tree composition sampled in the same country). The random factors included were (i) forest site identity (i.e., Country), (ii) unique identifier for each plot (i.e., Plot, to account for multiple measurements within each plot, namely that the observational units in each plot were the trees), (iii) identity of the tree species (i.e., Species, as species was crossed with country and composition and partially crossed with plot), (iv) tree species composition of each plot (i.e., Composition, as some plots have specific tree species compositions repeated, while others did not, coded as two‐letter combinations of species names), and (v) the interaction between tree species and plot identities, given that there were multiple measurements per species per plot. The first four random factors were necessary because of the experimental design. An additional random factor (i.e., number 5, the interaction between species and plot identities), which was not imposed by the design, was tested; the susceptibility of a tree species may differ depending on plot location or composition. To determine whether the interaction between tree species and plot identities contributed to the model, model comparison based on restricted maximum likelihood (REML) was used (Zuur et al. 2009). The model that included this interaction term was better than that without, and thus model selection for fixed effects was carried out with all random factors, including the interaction term.

Tree diversity and latitude were explanatory variables that were tested to determine the relationship between tree species diversity and fungal disease incidence across the latitudinal gradient. Tree diversity was further specified as tree species richness (Richness, 1–5 species), mean phylogenetic distance (MPD) among associated tree species, or functional type (FxnID) of the focal tree species, (i.e., broadleaved or conifer). The continuous variables included Richness, MPD, and Latitude. Richness was defined as 1‐ (monoculture), 2‐, 3‐, 4‐, or 5‐species mixture. Evenness among species abundance was a criterion when establishing mixed plots. As a result, tree species richness and Shannon's diversity index, based on basal area of focal trees, were highly correlated (*r* = 0.93). Results presented would have been qualitatively the same with the Shannon's diversity index (not shown). Mean phylogenetic distance (MPD) represented the mean of pairwise distances between associated tree species in a given plot. To calculate MPD, first a phylogenetic tree including all the tree species present in the six countries was computed using Phylomatic (Webb et al. 2008) and the APG III megatree (Bremer et al. 2009). Branch lengths (My) were added to the phylogeny using the BLADJ algorithm in Phylocom (Webb et al. 2008) and were based on the node ages from Wikstrom et al. (2001) and Crisp and Cook (2011). MPD was calculated with the R function *comdist* in the *picante* package (Kembel et al. 2010). Branch length was weighted by species basal area that accounted for unbalanced abundances of trees within plots. MPD was set to zero in monocultures. MPD was square‐root‐transformed (sqMPD) in order to account for the nonlinear relationship between evolutionary patterns and ecological processes (Letten and Cornwell 2015). The latitude of each plot in each country was explicitly specified. For graphical purposes and to visualize country‐specific trends, analyses were carried out again where the mean latitude for each country was instead specified and ranked from south to north.

To determine whether the explanatory variables were associated or correlated with Richness, we performed a Kruskal–Wallis rank‐sum test between categorical and continuous variables and Pearson's correlation between two continuous variables. The raw values for Richness ranged between 1 and 5, for sqMPD, between 0 and 26, and for Latitude, between 40°N and 63°N. Richness, sqMPD, and Latitude were scaled and centered to allow comparing coefficients and interpretation of simple effects of variables and their interactions (Schielzeth 2010).

Disease incidence was analyzed with the following fixed explanatory variables, with the full model including (i) Richness, (ii) sqMPD, (iii) Latitude, (iv) FxnID, and (v) the pairwise interactions between each of these explanatory variables. Model parameter estimates reported in the Results section for FxnID correspond to the reference level “broadleaved.”

Multiple regression models were constructed, in addition to the null model (i.e., intercept only), to test the effect of each explanatory variable. The set of best‐fitting models was selected based on Akaike's information criterion (AIC), corrected for small sample sizes (AICc) (Burnham and Anderson 2002). Among the best‐fitting models, the minimum adequate model, that is, most parsimonious model, was that with the lowest number of estimable parameters (*K*) within 2 AICc units (Δ_*i*_) of the model with the lowest AICc. The marginal coefficient of determination (*R* ^2^ m) for GLMMs is the variance explained by fixed factors (Nakagawa and Schielzeth 2013). The variance of the random‐effect terms and estimates of fixed‐effect terms, and their significance, were obtained for the model with the lowest AICc.

All analyses were carried out in R version 3.1.3 (R Core Team 2013). GLMMs were run with the *glmer* function, with the binomial distribution specified and logit link (logit = ln(number of diseased leaves/number of diseased and healthy leaves), in *lme4* (Bates et al. 2014). To aid in model convergence, default parameters were modified to include the optimizer “bobyqa” for both the preliminary and final steps, and the number of function evaluations was increased to 200,000. Model comparison was made with *selMod* in *pgirmess* (Giraudoux 2012). The *MuMIn* package was used to calculate AICc weights (*w*_*i*_) (function *importance*), which, for any explanatory variable, is calculated as the sum of *w*_*i*_ of all models that include this variable and explains the probability that a predictor is included in the best model and *R*² (function *r.squaredGLMM*) (Bartoń 2015).

## Results

**Figure 2:**
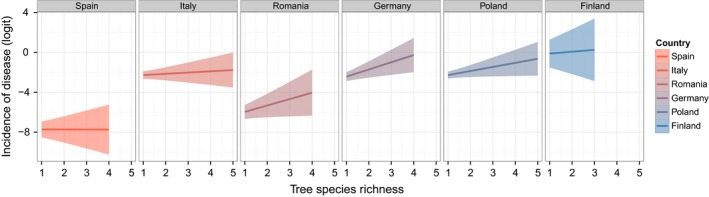
Predicted relationship between incidence of foliar disease and tree species richness across mature European forests. For these country‐specific analyses, the GLMM was modified for graphical visualization purposes that included removal of tree functional type (FxnID) from the model, and the mean latitude for each country was used, with countries ranked from south to north. The model is Incidence of disease ~ Richness + Country + Richness:Country + (Richness|Composition) + (1|Species:Plot). The predicted incidence of disease (solid line) on the logit scale (i.e., ln(number of diseased leaves/number of diseased and healthy leaves)) was computed for each country. Higher logit values correspond to higher incidence of disease. The shaded area shows the corresponding confidence interval.

The highest incidence of diseased leaves and shoots was detected in trees in Finland, while the lowest occurred in Spain and Romania (Fig. 2, Table S1). The percent of damaged leaves and shoots in Spain was between 0.7 and 3.1% (Table S1), and it ranked between 40.5 and 41.4% in Finland (Fig. 2, Table S1). The major fungal pathogens detected included *Chrysomyxa ledi* de Bary [rust] infecting *Picea abies* (L.) H.Karst. in Finland, *Rhytisma acerinum* Schwein. [leaf spots] on *Acer pseudoplatanus* L. in Germany and Romania, and *Erysiphe* sp. [oak powdery mildew] on *Quercus petraea* (Mattuschka) Liebl. and *Quercus robur* L. in Germany and Poland, respectively.

The explanatory variables square‐root‐transformed mean phylogenetic distance (sqMPD) and Latitude were not correlated with tree species richness (Richness). Richness and tree functional type (FxnID) were not fully independent (Kruskal–Wallis, *χ* ^2^ = 30.36, *P* < 0.001), but was retained in the final model despite the likely inflation in variance of model parameter estimates. The best model explaining fungal disease incidence included Richness, FxnID, Latitude, Richness × FxnID, and Richness × Latitude interactions as predictors (Table 2). Furthermore, these predictors had more than 99% chance of being retained in the best model (AICc weights >0.99). In contrast, sqMPD had 39% chance, and other pairwise interactions and those interactions that include sqMPD had between 0 and 16% chance of being retained.

**Table 2:** Results of model selection for analyses of foliar fungal disease incidence in European forests

Model	*K*	AICc	Δ_*i*_	*w*_*i*_	*R* ^2^ m
**Richness + FxnID + Latitude + Richness:FxnID + Richness:Latitude**	**11**	**8188.88**	**0**	**0.61**	**0.59**
Richness + FxnID + Latitude + sqMPD + Richness:FxnID + Richness:Latitude	12	8190.89	2.01	0.22	0.52
Richness + FxnID + Latitude + sqMPD + Richness:FxnID + FxnID:Latitude + Richness:Latitude	13	8192.53	3.65	0.1	0.59
Richness + FxnID + Latitude + sqMPD + Richness:FxnID + FxnID:Latitude + FxnID:sqMPD + Richness:Latitude	14	8194.55	5.67	0.04	0.59
Richness + FxnID + Latitude + sqMPD + Richness:FxnID + FxnID:Latitude + FxnID:sqMPD + Richness:Latitude + Richness:sqMPD	15	8196.15	7.27	0.02	0.58
Richness + FxnID + Latitude + sqMPD + Richness:FxnID + FxnID:Latitude + FxnID:sqMPD + Richness:Latitude + Richness:sqMPD + sqMPD:Latitude	16	8198.07	9.20	0.01	0.58
Richness + FxnID + Latitude + sqMPD + Richness:FxnID	11	8199.93	11.05	0	0.57
Richness + FxnID + Latitude + sqMPD + Richness:FxnID + FxnID:Latitude	12	8201.73	12.86	0	0.58
Richness + FxnID + Latitude + sqMPD	10	8203.66	14.79	0	0.56
Richness + FxnID + Latitude + sqMPD + Richness:FxnID + FxnID:Latitude + FxnID:sqMPD	13	8203.77	14.89	0	0.58
Null	6	8224.19	35.31	0	0

All models included the following random factors: country identity, plot composition, plot identity, tree species identity, and tree species identity × plot identity interaction (i.e., Country, Composition, Plot, Species, and Species x Plot, respectively). Multiple regression models are shown, including their number of estimable parameters (*K*), and their Akaike weights (*w*_*i*_), the relative likelihood of the model. Model within 2 AICc units (Δ_*i*_) of the model with the lowest AICc is bolded. Marginal coefficient (*R* ^2^ m), which is the variance explained by fixed factors, is indicated.

Null = null model (intercept only); Richness = tree species richness; FxnID = functional type of the tree species (i.e., broadleaved or conifer); Latitude = explicitly specified for each plot; sqMPD = square‐root‐transformed mean phylogenetic distance.

The model parameter estimate for the Richness × Latitude interaction was significantly positive (0.35 ± 0.10, Table S2). The relationship between tree species richness and disease incidence increased with the increase in latitude. Graphical visualization of the tendencies for each country indicated that there was a general increase, although non‐significant, in disease incidence with Richness in Romania, Germany, Poland, and Finland (Fig. 2, Table S3).

**Figure 3:**
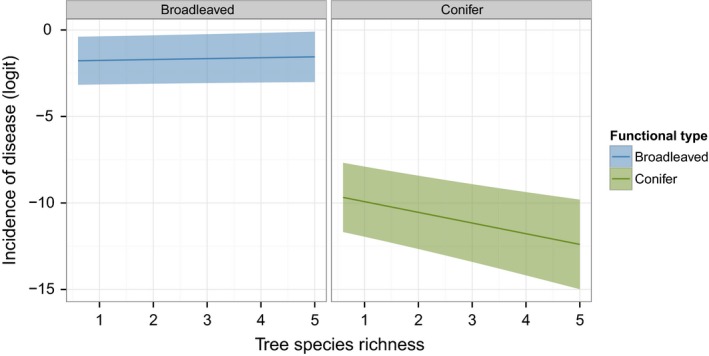
Predicted relationship between the incidence of foliar disease of broadleaved and conifer tree species and tree species richness (Richness) in mature European forests. The predicted incidence of disease (solid line) on the logit scale (i.e., ln(number of diseased leaves/number of diseased and healthy leaves)) was computed for each tree functional type (i.e., broadleaved or conifer). For these tree functional type‐specific analyses, the GLMM with the lowest AICc was simplified by the removal of latitude from the model. The model is Incidence of disease ~ Richness + FxnID + Richness:FxnID + (1|Country) + (1|Composition) + (1|Plot) + (1|Species) + (1|Species:Plot). The shaded area shows the corresponding confidence interval.

The model parameter estimate for the Richness × FxnID interaction was significantly negative (−1.05 ± 0.33, see Table S2). For conifer species, foliar damage tended to decrease with Richness, while there was a tendency to increase with Richness for broadleaved species (Fig. 3 and parameter estimate for conifers −0.67 ± 0.31, Table S4).

Variance partitioning of the random effects indicated that most of the variance was explained by the identity of tree species, while the other factors (country identity, plot identity, and plot composition) accounted for very small amounts (Table S2). There was also a non‐zero variance of the tree species identity × plot identity interaction, which may be explained by the same species being considered a different factor in different plots, where the susceptibility of a species can change depending on the species composition of the plot.

The relationship between disease incidence and tree diversity variables, Richness and FxnID, was also tested in individual countries to remove the effect of Latitude and Richness × Latitude. In Spain, Italy, and Romania, there was no relationship between disease incidence and Richness and/or FxnID (data not shown). A relationship between disease incidence and FxnID was observed in Poland (−7.21 ± 1.16, *P* < 0.05) and Finland (−10.06 ± 4.66, *P* < 0.05), indicating reduced disease incidence for conifers relative to broadleaved species. A relationship between disease incidence and Richness was also observed in Germany (0.45 ± 0.18, *P* < 0.05), indicating increased disease incidence with increasing Richness. Additionally, the disease incidence for specific fungal pathogens was analyzed for *C. ledi* in Finland, for *R. acerinum* in Romania, as well as for *Erysiphe* sp. in Germany and Poland. No significant relationship between disease incidence and Richness was found.

## Discussion

Our study integrated a broad range of different mature forest ecosystems across a continental scale to understand the relationship between tree diversity and the incidence of foliar fungal disease. Across six European forest types, there were complex interactions between tree species richness and latitude, and tree species richness and tree functional type that together determined the disease incidence on leaves and shoots of mature trees.

For a majority of forests assessed in this study, there was a general albeit non‐significant trend for increasing disease incidence with increasing tree species richness. This is in contrast to earlier theories of decreased biotic damages with increasing host diversity (Heybroek 1982; Keesing et al. 2006; Jactel and Brockerhoff 2007), and may suggest that tree species experience associational susceptibility to foliar fungal pathogen infestation in higher diversity mixtures (Barbosa et al. 2009). Hantsch et al. (2014a) demonstrated that while associational resistance to fungal pathogens was observed at the tree species level, neither associational resistance or susceptibility was a general phenomenon. Earlier studies on pathogenic root fungi found that disease risk was reduced in mixed forest stands or when the proportion of host trees was reduced (Lindén and Vollbrecht 2002; Thor et al. 2005). However, patterns may be different for airborne pathogens of mature trees.

Associational susceptibility to foliar fungal pathogen infestation may be the result of different ecological mechanisms. Mixed forests may provide more suitable microclimates for fungi (e.g., higher humidity) than monocultures (Lodge and Cantrell 1995; Jules et al. 2014). Trees in mixture have been known to compete with one another and may affect the overall fitness of susceptible tree species (Pollastrini et al. 2013). Furthermore, the predominance of generalist pathogen species that can spill over from one host tree to another could increase the inoculum, and consequently disease in mixtures (Maloney et al. 2005; Parker et al. 2015).

The relationship between tree species richness and fungal disease incidence also depended on the functional type of the tree species, specifically, whether the tree was a broadleaved or conifer species. Increasing tree species richness decreased fungal pathogen damages for conifer species, but slightly increased damages for broadleaved species. Such contrasting response between conifer and broadleaved species to fungal pathogen damage has not been previously reported. One important factor may be that conifers retain their needles for several years. These can thus serve as an inoculum source for within plot spread. The leaves of deciduous trees would be on the forest floor the following year and in less favorable position for any spore dispersal. Furthermore, the differences may result from specific leaf traits inherent for the tree species (Valkama et al. 2005). Some broadleaved species have higher polyphenolic content in their leaves that correlated with increased pathogen richness, compared to conifers that were uninfected by pathogens (Hantsch et al. 2014b). Tree diversity can modify these traits by influencing the fitness of tree species (Haase et al. 2015).

However, the observed pattern needs to be interpreted with caution, as it may reflect a species‐specific effect rather than a general tendency. Little‐to‐no damages of conifers were detected in Spain, Romania, Germany, and Poland. Only *P. abies* in Finland was diseased. Thus, the pattern of decreasing damages with tree species richness observed for conifers is mainly driven by damages observed in Finland on *P. abies*. Analyses conducted in Finland alone indicated that *P. abies* had decreased disease incidence compared to the broadleaved species, *Betula pendula*, although no significant relationship with tree species richness was observed (data not shown). It is highly likely that the inoculum source for the main disease of conifer needle disease in Finland, *C. ledi*, would be from the alternating host outside the plot.

The relationship between mean phylogenetic distance (sqMPD) and disease incidence could not be established. Phylogenetic diversity among tree species is expected to decrease the level of pathogen spillover; the more evolutionary distant the host trees are, the less likely they share common pathogens (Gilbert and Webb 2007; Castagneyrol et al. 2014; Parker et al. 2015). In this study, mixing conifers and broadleaved species, or more generally closely and remotely phylogenetically related species, did not affect pathogen damages. However, tree species identity may play a larger role (McCracken and Dawson 2003; Lamit et al. 2015). There seems to be other factors specific to individual tree species that influenced overall disease incidence, as evidenced by the high variance attributed to the random factor tree species identity (i.e., Species). Tree species‐specific disease incidence patterns are likely variable and depend on seasonal and interannual variation. These variations influence host nutrition and defense response and the ability of fungal pathogens to germinate, disperse, and infect (Lodge and Cantrell 1995; Camarero et al. 2015). Additionally, there was a non‐null variance of species within plots, suggesting that species‐specific behavior regarding disease incidence may be influenced by some landscape factor. This may contribute to the lack of effect of sqMPD, which assumes that tree traits are fixed. Traits may vary with trait neighborhood, and depending on the magnitude of this neighbor‐related trait variability, it may explain why MPD was not that important.

Landscape features can influence the spread of pathogens and expression of disease (Holdenrieder et al. 2004; Haas et al. 2011), in particular because spores can spread over large distances (Zeglen 2002). The major pathogen recorded in this study on conifers was the spruce needle rust (*C. ledi* infecting *P. abies*). This rust requires the presence of the alternate host, the shrub *Rhododendron tomentosum* Harmaja (syn. *Ledum palustre* L.) to complete its life cycle. This shrub has not been detected within the sampling plots in Finland (E. Ampoorter, personal communication), but the observed disease pattern could have been the result of the presence of the alternate host in the surrounding landscape.

The observed differences in fungal disease incidence patterns across latitudes and between tree functional types may be a result of landscape effects and the timing of sampling. Tree diversity should also be considered at the landscape level. There was a statistically significant increase in disease incidence with increasing latitude. Latitude is a complex ecological factor commonly used as a proxy for other factors changing in the environment, especially temperature and precipitation, both of which have been shown to be important for pathogen richness (Vacher et al. 2008). Temperature and moisture optima of fungal pathogens influence their ability to germinate, grow, sporulate, and infect or be present in an environment (Oliva et al. 2013). The higher disease incidence in Finland compared to Spain may thus be explained by differences in conducive environmental conditions such as humidity. This could be tested by considering the same tree species, for example *Pinus sylvestris*, present in both Finland and Spain. In our study, we, however, found no disease on *P. sylvestris* in either country.

Time of sampling is also important for the detection of foliar fungal pathogen damages. The sample collection scheme in this study aimed to sample each country in the period when trees were “phenologically equivalent.” However, this was not always achieved. Low levels of fungal pathogen damages in the Mediterranean countries, specifically in Spain, may be attributed to a too early sampling of the broadleaved species during the vegetation season when leaves had recently flushed (e.g., *Quercus faginea* flushed approximately 2 weeks before sampling). Leaves would be expected to express more symptoms later in the season overall and specifically following drought stress (Jactel et al. 2012). Additionally, too early sampling would underestimate the presence and severity of infection. For instance, at the time of sampling in Romania, *Rhytisma* tar spots were rather small (<2 mm in diameter) and could easily have been missed or misidentified as damages caused by other abiotic or nonfungal biotic agents. Furthermore, conifers efficiently shed their needles following infection. Sampling after needle shed would underestimate the presence and severity of damages. Thus, timing of sampling needs to be carefully considered to balance, on the one hand, sufficient development of damage symptoms on foliage and, on the other, leaf shed following infection, for the specific pathogens one expects to find in specific tree species.

In conclusion, we found foliar fungal disease incidence to increase with tree species richness in mature forests, although the magnitude of this effect seems to vary along the latitudinal gradient and between tree functional types. Several environmental factors may have obscured more general trends; one such factor includes landscape effects, not yet captured in our study design. Our results call for further studies to elucidate the important specific drivers of foliar fungal pathogen incidence in mature forest ecosystems.

## Data Accessibility

The fungal disease incidence data used for this publication have been archived in Dryad. DOI:10.5061/dryad.389mt. The R script used for analyses can be found as online supporting information.

## Conflict of Interest

None declared.

## Supporting information


**Table S1.** Incidence of diseased leaves and shoots by country and richness levels within each country, represented as the percent disease incidence and the corresponding logit (= Percent damage/100‐Percent damage).
**Table S2.** Model parameter estimates of the overall model with the lowest AICc for the incidence of foliar fungal disease in European forests.
**Table S3.** Model parameter estimates of the modified random slope, random intercept model for the incidence of foliar fungal disease along a tree species richness gradient in European forests in each country.
**Table S4.** Model parameter estimates of the modified model for the incidence of foliar fungal disease along a tree species richness gradient for broadleaved and conifer trees in European forests.

**Appendix S1.** R script.
