# Supplementary material for: Fungal disease incidence along tree diversity gradients depends on latitude in European forests
Source: Ecol Evol. 2016 Mar 11;6(8):2426–38. doi: 10.1002/ece3.2056 (PMC4788975; doi:10.1002/ece3.2056)
Supplement: Supplementary file 1 — Table S1. Incidence of diseased leaves and shoots by country and richness levels within each country, represented as the percent disease incidence and the corresponding logit (= Percent damage/100‐Percent damage). Table S2. Model parameter estimates of the overall model with the lowest AICc for the incidence of foliar fungal disease in European forests. Table S3. Model parameter estimates of the modified random slope, random intercept model for the incidence of foliar fungal disease along a tree species richness gradient in European forests in each country. Table S4. Model parameter estimates of the modified model for the incidence of foliar fungal disease along a tree species richness gradient for broadleaved and conifer trees in European forests. [file ECE3-6-2426-s001.docx]

**Supporting Information**

Additional Supporting Information may be found in the online version of this article:

**Appendix S1.** R scripts.

**Table S1.** Incidence of diseased leaves and shoots by country and richness levels within each country, represented as the percent damage and the corresponding logit (= Percent damage/100-Percent damage).

**Table S2.** Model parameter estimates of the overall model with the lowest AICc for the incidence of foliar fungal disease in European forests.

**Table S3.** Model parameter estimates of the modified random slope, random intercept model for the incidence of foliar fungal disease along a tree species richness gradient in European forests in each country.

**Table S4.** Model parameter estimates of the modified model for the incidence of foliar fungal disease along a tree species richness gradient for broadleaved and conifer trees in European forests.

**Table S1.** Incidence of diseased leaves and shoots by country and richness levels within each country, represented as the percent damage and the corresponding logit (= Percent damage/100-Percent damage)

| Country | Richness | Diseased foliage | Total foliage | Percent damage | Logit |
| --- | --- | --- | --- | --- | --- |
| Spain |  |  |  |  |  |
|  | 1 | 132 | 2877 | 3.1 | -3.44 |
|  | 2 | 101 | 3595 | 1.9 | -3.96 |
|  | 3 | 61 | 2159 | 1.9 | -3.95 |
|  | 4 | 16 | 1438 | 0.7 | -4.90 |
|  | **Total** | **310** | **10069** |  |  |
| Italy |  |  |  |  |  |
|  | 1 | 1000 | 3414 | 28.6 | -0.91 |
|  | 2 | 680 | 2937 | 23.8 | -1.17 |
|  | 3 | 679 | 4272 | 15.9 | -1.67 |
|  | 4 | 1009 | 5080 | 19.6 | -1.41 |
|  | 5 | 105 | 900 | 11.7 | -2.02 |
|  | **Total** | **3473** | **16603** |  |  |
| Romania |  |  |  |  |  |
|  | 1 | 50 | 1871 | 1.8 | -3.99 |
|  | 2 | 45 | 2395 | 1.3 | -4.37 |
|  | 3 | 76 | 2536 | 2.3 | -3.75 |
|  | 4 | 57 | 1395 | 2.7 | -3.58 |
|  | **Total** | **228** | **8197** |  |  |
| Germany |  |  |  |  |  |
|  | 1 | 192 | 1445 | 12.0 | -1.99 |
|  | 2 | 374 | 2810 | 12.3 | -1.97 |
|  | 3 | 895 | 6701 | 12.7 | -1.92 |
|  | 4 | 699 | 2850 | 23.0 | -1.21 |
|  | **Total** | **2160** | **13806** |  |  |
| Poland |  |  |  |  |  |
|  | 1 | 463 | 1900 | 16.1 | -1.65 |
|  | 2 | 1340 | 3420 | 29.8 | -0.86 |
|  | 3 | 2029 | 4738 | 32.6 | -0.73 |
|  | 4 | 2127 | 5280 | 29.5 | -0.87 |
|  | 5 | 604 | 1320 | 33.6 | -0.68 |
|  | **Total** | **6563** | **16658** |  |  |
| Finland |  |  |  |  |  |
|  | 1 | 1295 | 2160 | 40.5 | -0.38 |
|  | 2 | 1291 | 2160 | 40.8 | -0.37 |
|  | 3 | 631 | 1055 | 41.4 | -0.35 |
|  | **Total** | **3217** | **5375** |  |  |

**Table S2.** Model parameter estimates of the overall model with the lowest AICc for the incidence of foliar fungal disease in European forests

| **Random effects** | |  |  |  |  |
| --- | --- | --- | --- | --- | --- |
|  |  | Variance | StdDev |  |  |
|  | Composition | < 0.01 | < 0.01 |  |  |
|  | Plot | 0.23 | 0.48 |  |  |
|  | Country | 0.29 | 0.54 |  |  |
|  | Species:Plot | 1.05 | 1.02 |  |  |
|  | Species | 6.82 | 2.61 |  |  |
|  |  |  |  |  |  |
| **Fixed effects** | |  |  |  |  |
|  |  | Estimate | Std.Error | z value | *P* (>\|z\|) |
|  | Intercept^#^ | -1.87 | 0.80 | -2.34 | 0.019 |
|  | Richness | 0.12 | 0.09 | 1.38 | 0.167 |
|  | FxnID* | -7.81 | 1.74 | -4.49 | < 0.001 |
|  | Latitude† | 2.68 | 0.29 | 9.17 | < 0.001 |
|  | Richness:FxnID* | -1.05 | 0.33 | -3.17 | 0.002 |
|  | Richness:Latitude† | 0.35 | 0.10 | 3.35 | 0.001 |
| ^#^ The intercept is for broadleaved trees at Richness level 1, the monoculture.  * FxnID= functional type of the tree species (i.e. broadleaved or conifer). Model parameter estimates for FxnID correspond to the reference level “broadleaved”.  † Latitude is a continuous variable with the latitude of each plot explicitly specified. | | | | | |

**Table S3.** Model parameter estimates of the modified random slope, random intercept model for the incidence of foliar fungal disease along a tree species richness gradient in European forests in each country

| **Random effects** | |  |  |  |  |
| --- | --- | --- | --- | --- | --- |
|  |  | Variance | StdDev | Corr |  |
|  | Species:Plot | 7.42 | 2.72 |  |  |
|  | Composition | 10.76 | 3.28 |  |  |
|  | Richness | 0.75 | 0.86 | -1 |  |
|  |  |  |  |  |  |
| **Fixed effects** | |  |  |  |  |
|  |  | Estimate | Std.Error | z value | *P* (>\|z\|) |
|  | Intercept* | -0.75 | 1.87 | -0.40 | 0.69 |
|  | Richness | 0.17 | 0.81 | 0.21 | 0.83 |
|  | Spain | -6.96 | 2.47 | -2.82 | <0.01 |
|  | Italy | -1.94 | 2.20 | -0.88 | 0.38 |
|  | Romania | -7.35 | 2.36 | -3.11 | <0.01 |
|  | Germany | -4.08 | 2.15 | -1.90 | 0.06 |
|  | Poland | -2.88 | 2.01 | -1.43 | 0.15 |
|  | Richness:Spain | -0.17 | 0.98 | -0.18 | 0.86 |
|  | Richness:Italy | -0.06 | 0.88 | -0.06 | 0.95 |
|  | Richness:Romania | 0.41 | 0.94 | 0.43 | 0.67 |
|  | Richness:Germany | 0.48 | 0.87 | 0.55 | 0.58 |
|  | Richness:Poland | 0.20 | 0.84 | 0.23 | 0.82 |
| * The intercept is for Finland at Richness level 1, the monoculture. | | | | | |

**Table S4.** Model parameter estimates of the modified model for the incidence of foliar fungal disease along a tree species richness gradient for broadleaved and conifer trees in European forests

| **Random effects** | |  |  |  |  |
| --- | --- | --- | --- | --- | --- |
|  |  | Variance | StdDev |  |  |
|  | Composition | 0.04 | 0.19 |  |  |
|  | Plot | 0.24 | 0.49 |  |  |
|  | Country | 7.68 | 2.77 |  |  |
|  | Species:Plot | 1.05 | 1.02 |  |  |
|  | Species | 7.34 | 2.71 |  |  |
|  |  |  |  |  |  |
| **Fixed effects** | |  |  |  |  |
|  |  | Estimate | Std.Error | z value | *P* (>\|z\|) |
|  | Intercept^#^ | -1.81 | 1.39 | -1.30 | 0.19 |
|  | Richness | 0.05 | 0.09 | 0.58 | 0.56 |
|  | FxnID* | -7.50 | 1.80 | -4.16 | <0.001 |
|  | Richness:FxnID* | -0.67 | 0.31 | -2.17 | 0.03 |
| ^#^ The intercept is for broadleaved trees at Richness level 1, the monoculture.  * FxnID= functional type of the tree species (i.e. broadleaved or conifer). Model parameter estimates for FxnID correspond to the reference level “broadleaved”. | | | | | |
